# The Metabolic Sensor GPR43 Receptor Plays a Role in the Control of *Klebsiella pneumoniae* Infection in the Lung

**DOI:** 10.3389/fimmu.2018.00142

**Published:** 2018-02-20

**Authors:** Izabela Galvão, Luciana P. Tavares, Renan O. Corrêa, José Luís Fachi, Vitor Melo Rocha, Marcela Rungue, Cristiana C. Garcia, Geovanni Cassali, Caroline M. Ferreira, Flaviano S. Martins, Sergio C. Oliveira, Charles R. Mackay, Mauro M. Teixeira, Marco Aurélio R. Vinolo, Angélica T. Vieira

**Affiliations:** ^1^Department of Biochemistry and Immunology, Institute of Biological Sciences, Federal University of Minas Gerais, Belo Horizonte, Brazil; ^2^Department of Genetics, Evolution and Bioagents, Institute of Biology, University of Campinas, Campinas, Brazil; ^3^Laboratory of Respiratory Viruses and Measles, Oswaldo Cruz Institute, Fiocruz, Rio de Janeiro, Brazil; ^4^Department of General Pathology, Institute of Biological Sciences, Federal University of Minas Gerais, Belo Horizonte, Brazil; ^5^Department of Pharmaceutics Sciences, Institute of Environmental, Chemistry and Pharmaceutical Sciences, Universidade Federal de São Paulo, Diadema, Brazil; ^6^Department of Microbiology, Institute of Biological Sciences, Federal University of Minas Gerais, Belo Horizonte, Brazil; ^7^Department of Immunology, Monash University, Melborne, VIC, Australia

**Keywords:** lung infection, GPR43, inflammation, microbiota, short-chain fatty acids, pneumonia

## Abstract

Pneumonia is one of the leading causes of death and mortality worldwide. The inflammatory responses that follow respiratory infections are protective leading to pathogen clearance but can also be deleterious if unregulated. The microbiota is known to be an important protective barrier against infections, mediating both direct inhibitory effects against the potential pathogen and also regulating the immune responses contributing to a proper clearance of the pathogen and return to homeostasis. GPR43 is one receptor for acetate, a microbiota metabolite shown to induce and to regulate important immune functions. Here, we addressed the role of GPR43 signaling during pulmonary bacterial infections. We have shown for the first time that the absence of GPR43 leads to increased susceptibility to *Klebsiella pneumoniae* infection, which was associated to both uncontrolled proliferation of bacteria and to increased inflammatory response. Mechanistically, we showed that GPR43 expression especially in neutrophils and alveolar macrophages is important for bacterial phagocytosis and killing. In addition, treatment with the GPR43 ligand, acetate, is protective during bacterial lung infection. This was associated to reduction in the number of bacteria in the airways and to the control of the inflammatory responses. Altogether, GPR43 plays an important role in the “gut–lung axis” as a sensor of the host gut microbiota activity through acetate binding promoting a proper immune response in the lungs.

## Introduction

Pneumonia remains a leading cause of death and hospitalization worldwide, especially among children and the elderly. It is estimated that more than 150 million cases occur annually among children under 5 years old, leading to approximately 2 million deaths each year (1). Pneumonia is a lower respiratory tract disease characterized by an intense inflammatory response, which results in increased lung edema, loss of respiratory area, and can lead to respiratory impairment and death (2–4). It can be caused by several pathogens, such as virus, fungi, and bacteria. Among bacterial causes of pneumonia, *Streptococcus pneumoniae* and *Klebsiella pneumoniae* (*Kp*) are the main causes of community-acquired pneumonia or nosocomial pneumonia, respectively (5, 6).

During respiratory tract infections, the inflammatory response is very important to control pathogen replication and dissemination; however, the inflammation itself could be harmful for the host as a collateral damage (7). The overproduction of cytokines and chemokines and the overactivation of leukocytes, especially neutrophils, can lead to an intense lung injury. Therefore, the lungs have several mechanisms to assure an effective clearance of potential pathogens and to restrain the overwhelming inflammation that could lead to a worse outcome (8). In this regard, a community of indigenous microorganisms composing the host microbiota is important to guarantee the balance between proper immune response and minimum tissue damage (9, 10). The host microbiota can exert its actions through both direct and indirect effects, such as by the production of short-chain fatty acids (SCFA) (11). Those microbial metabolites are not only nutrients, being used as an energy source for colonocytes, but also act as signaling molecules. Therefore, SCFA were shown to be important regulating crucial host cellular processes such as adipogenesis, intestinal function, and also inflammation (12). Indeed, SCFA were shown to increase some important functions of leukocytes, such as production of reactive oxygen species (13), but also to downregulate the production of pro-inflammatory cytokines and the magnitude of neutrophilic inflammation in several disease models (14).

The majority of SCFA produced by microbiota fermentation are acetate, propionate, and butyrate. Those molecules can act by epigenetic changes due to inhibition of histone deacetylase and through binding and activation of orphan G-protein-coupled receptors, such as GPR41, GPR43, and GPR109 (15). GPR43 binds and gets activated specially by acetate, and it is considered an important dietary nutritional sensor (16, 17). Because gut microbiota is the main source of SCFA and is very sensitive to nutritional alterations (18), changes in microbiota reflect a decreased SCFA production and GPR43 activation. This receptor is highly expressed in inflammatory cells, such as neutrophils and macrophages (MF) (11), therefore sensing the nutritional status of the host and regulating important functional aspects in immune responses (19). GPR43 was shown to be important for the production of intestinal IgA (20) and also for the development of inflammatory response during different diseases, such as asthma (19), gout (13), and colitis (21). In this regard, the presence of gut microbiota and sensing of their metabolites through GPR43 leads to a modulation of local and systemic inflammatory responses important to the mucosal tissues.

Despite its involvement in several inflammatory diseases, the role of GPR43 during lung infections has never been addressed. Therefore, we showed here, for the first time, the importance of GPR43 during respiratory infections being this receptor important for both clearance of bacteria and most importantly for the regulation of immune responses, which could lead to lung damage during infections.

## Materials and Methods

### Mice

GPR43/FFAR2 gene-deficient (Gpr43^−/−^) mice were produced as previously described (11) backcrossed onto the C57BL/6 background for 10 generations, and maintained in the animal facilities at Universidade Federal de Minas Gerais (UFMG; Belo Horizonte, Brazil). Wild-type male C57BL/6 mice (8–12 weeks) were obtained from the centre of animal care (CEBIO) from UFMG. Mice were maintained in pathogen-free and co-housing conditions with free access to filtered water and food. This study was carried out in accordance with the recommendations of the local animal ethics committee at Universidade Federal de Minas Gerais (CETEA/UFMG 219/2014).

### Pulmonary Infection

*Klebsiella pneumoniae* (ATCC 27736) lung infection was made as previously described (22). Briefly, animals were anesthetized i.p. with 0.2 mL of a solution containing xylazine (02 mg/mL), ketamine (50 mg/mL), and saline in a proposition of 1:0.5:3. The trachea was exposed and 25 µL of a suspension containing 1 × 10^6^ CFU of *Kp* or saline was administered with a 26-gauge needle. After the infection, the animals were euthanized at different time points.

*Streptococcus pneumoniae* (ATCC 6303 serotype 3) (*Sp*) infection was made as previously described (7). Briefly, mice were instilled intranasally with 10^5^ or 10^6^ CFU of *Sp* in 40 µL and were euthanized at 48 h post infection. The data were not shown, and it is only mentioned in the Section “Result.”

### Acetate Treatment

Mice were given sodium acetate (Sigma-Aldrich, St. Louis MO, USA) for 5 days before the infection until they were killed. Acetate was added in the sterile drinking water of mice *ad libitum* at 150 mM. The acetate dose was chosen according previous data (11, 13), and pH was adjusted as needed. The water solution was changed every day to avoid contamination and significant changes.

### BALF and Tissue Extraction

Mice were euthanized with a lethal solution of ketamine/xylazine (180 and 12 mg/kg, respectively), and bronchoalveolar lavage (BAL) was performed by inserting and collecting three times 1 mL aliquots of phosphate-buffered saline (PBS), through a 1.7-mm catheter in a 1-mL syringe to quantify leukocytes recruited to the airways and bacteria counts. Total counts were performed by Neubauer chamber count. Differential counts were obtained from cytospin preparations. Lungs were collected for quantification of myeloperoxidase to assess neutrophil infiltration, cytokines determination, and histological examination of lung injury.

### Cytokine Determination

Tissues were collected and homogenized in PBS containing anti-proteases (22). Samples were centrifuged, and the supernatant was used for cytokine concentrations, in accordance with the manufacturer’s instructions (R&D Systems, Minneapolis, MN, USA).

### Histological Analysis

For histopathological analysis, lungs were removed and fixed for 24 h in 10% neutral buffered formalin (Sigma-Aldrich, St. Louis MO, USA). Samples were washed with PBS and dehydrated. After embedding in paraffin, 3-mm sections were obtained and stained with hematoxylin and eosin to allow assessment of inflammation in the lung. All lung sections, H&E-stained and at 100× magnification (11 mm^2^/field), were evaluated in a blinded manner. Two parameters were measured: severity of inflammation (0–3: none, slight, moderate, severe) and presence of edema (0–2: none, one-third damaged, and two-thirds damaged). The score of each parameter was multiplied by a factor reflecting the percentage of tissue involvement (1, 0–25%; 2, 26–50%; 3, 51–75%; and 4, 76–100%) and added to a sum. The maximum possible score is 20.

### Phagocytosis Assay

The phagocytosis assay was performed as previously described (23). Briefly, MF and neutrophils isolated from the bone marrow by gradient percoll separation were incubated in the presence of acetate (1 mM) and opsonized-pHRodo-marked *Kp* (MOI1:1). Cells [0.5 × 10^6^ for neutrophils and 0.5 × 10^5^ alveolar macrophage (AM)] were incubated in RPMI 1640 medium without antibiotics supplemented with 10% heat-inactivated FBS for 60 (AM) or 120 min (neutrophils). Cells were then washed, resuspended in 100 µL PBS, and incubated with anti-F4/80 (APC anti-mouse F4/80 Clone BM8, BioLegend, San Diego, CA, USA) and −CD45 (Pe-Cy7 anti-mouse CD45, BioLegend) or anti-Ly-6G (PE anti-mouse Ly-6G Clone 1A8, BioLegend). Trypan blue at a final concentration of 0.5% was added to quench the fluorescence of non-internalized bacteria. Negative controls consisting of cells incubated without *Kp* were used to set the fluorescence. Samples (10,000 events) were analyzed by flow cytometry (Gallios, Beckman Coulter, Brea, CA, USA). Both the percentage of cells that internalized bacteria and the mean fluorescence of bacteria within the cells were analyzed.

### Killing Assay of AM

Alveolar macrophage (0.5 × 10^5^) were incubated in the presence of acetate (1 mM) and opsonized *Kp*. Samples were incubated in RPMI 1640 medium without antibiotics supplemented with 10% heat-inactivated FBS for 30 or 120 min. Cells were centrifuged (1,500 rpm, 10 min, 4°C), and the supernatant was collected, serially diluted in sterile PBS (1/10, 1/1,000, and 1/100,000) and plated in triplicate at MacConkey agar. A 10-µL volume of undiluted bacteria and bacterial incubated in the absence of cells (total bacteria) were also plated as positive control. The plates were incubated in incubator at 37°C for 24 h. Colonies were then counted and the total number of CFU was obtained considering the dilution of the sample. The number of CFU was normalized considering the viable bacteria counted in the control condition (WT without treatment).

### Measurement of Cytokines Produced by Neutrophils and BMDM

Bone marrow isolated neutrophils (0.5 × 10^6^) and BMDM (1.5 × 10^5^) were incubated in medium RPMI 1640 without antibiotics and supplemented with 10% FBS in the presence or absence of *Kp* (MOI: 1:1) and acetate (1 mM) for 120 min. The supernatant of the cultures was collected and then assayed for cytokines (TNF-α, IL-1β, and IL-10) as described above.

### Neutrophil Chemotaxis Assay

Residents peritoneal MF were harvested from wild-type and Gpr43^−/−^ mice pretreated for 5 days or not with oral acetate (150 mM). Macrophages (1 × 10^6^ cells/well) were plated and stimulated with lipopolysaccharides (LPS) (1 µg/mL) for 24 h, and the supernatant was collected. Thioglycollate elicited neutrophils from wild-type and Gpr43^−/−^ were collected 18 h after intraperitoneal administration. Neutrophils were plated (1 × 10^5^ cells/well) on top of the filter membrane in a transwell insert and incubated for 10 min at 37°C and 5% CO_2_ to allow the cells to settle down. Macrophage supernatant was used as a chemoattractant into the bottom of the lower chamber in a 24-well plate. Cells were incubated for 30 min. The quantification of migrated cells was performed by count the migrated cells dropped into the media in the lower chamber. The number of migrated cells was counted by using flow cytometer and the confirmation by cytospin.

### AM Transference *In Vivo*

Wild-type mice were pretreated for 5 days with oral acetate (150 mM). Mice were euthanized with a lethal solution of ketamine/xylazine and BAL was performed by inserting and collecting three times 1 mL aliquots of PBS, through a 1.7-mm catheter in a 1-mL syringe. The isolation of AMs were performed as previously described (24). Briefly, AM were harvested from BAL and isolated by adherent cells to plastic for 1 h at 37 C in 5% CO_2_. The purity was confirmed by morphological analysis by cyotspin (92% of AM). For adoptive *in vivo* transfer AM were recovered by cold PBS and 2 × 10^5^ cells were instilled intranasally in wild-type and Gpr43^−/−^ mice 1 h before the *Kp* infection. 24 h after infection, mice were euthanized, and the BAL was performed. CFU and number of neutrophils were analyzed.

### Statistical Analyses

All results are presented as the means ± SEM. The analyses of the difference between two groups were performed by Student’s *t*-test. Normalized data of three or more groups were analyzed by one-way and two-way ANOVA, and differences between groups were assessed using the Newman–Keuls and Sidak’s posttest respectively. Results were considered significant when *P* ≤ 0.05. Calculations were performed using Prism 5.0 software for Windows (GraphPad Software, La Jolla, CA, USA).

## Results

### Gpr43^−/−^ Mice Are More Susceptible to *Kp* Lung Infection

**Figure 1:**
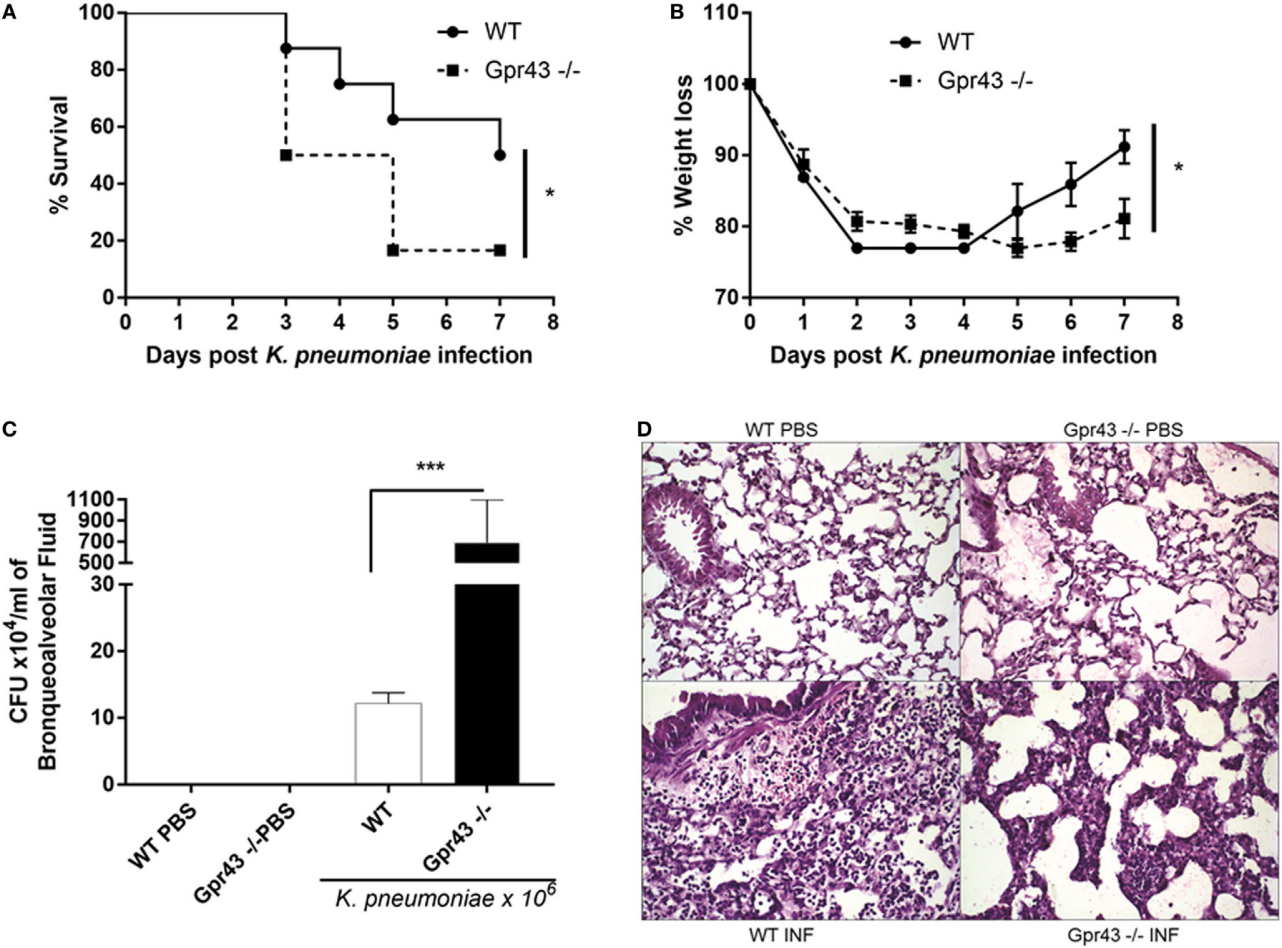
Gpr43^−/−^ mice are more susceptible to Gram-negative [*Klebsiella pneumoniae* (*Kp*)] lung infection. **(A)** Survival rate of mice were observed during 7 days following intratracheal *Kp* (1 × 10^6^ CFU). **(B)** Weight loss infection post-*Kp* infection. **(C)** Bacterial burden in the airways was assessed in bronchoalveolar fluid [bronchoalveolar lavage (BAL)] 48 h post-*Kp* infection. **(D)** Representative photographs of H&E-stained sections of lung from wild-type mice control [WT phosphate-buffered saline (PBS)], wild-type mice *Kp* infected (WT INF), Gpr43^−/−^ mice control (Gpr43^−/−^ PBS) and Gpr43^−/−^ mice infected (Gpr43^−/−^ INF). Scale bar—400 mm. Results are expressed as mean ± SEM of 8–10 mice per group. *Statistical difference (*P* < 0.05) when comparing to WT and Gpr43^−/−^ mice. ***Statistical difference (*P* < 0.001) when comparing to WT and Gpr43^−/−^ mice. Results representative of three independent experiments and the statistical (ANOVA—Posttest Newman–Keuls) was used.

Gpr43 is a receptor that binds to SCFA, particularly acetate, and is highly expressed on neutrophils and MF (11). To better understand the role of Gpr43 and its effects during infection, wild-type and Gpr43^−/−^ mice were infected with Gram-negative bacteria, *Kp*, by intratracheal challenge (1 × 10^6^ CFU). Mice weight and lethality were monitored for 7 days. Interestingly, Gpr43^−/−^ mice exhibited an increased susceptibility to infection, as observed by the higher mortality (Figure 1A) and by weight loss (Figure 1B) when compared to wild-type infected animals. These factors were associated to an increased burden of bacteria in the airways of Gpr43^−/−^ mice (Figure 1C). Histological analyses and score (WT PBS: 0; WT INF: 15.6 ± 1.15; GPR43^−/−^ PBS:0; GPR43^−/−^ INF:13.3 ± 2.3) showed a higher level of lung injury, which was observed by increased pulmonary edema and inflammatory cell infiltration in lung parenchyma and airways after 48 h of *Kp* infection in both Gpr43^−/−^ and wild-type mice (Figure 1D). Toward these results, we investigated whether the protective role of GPR43 was also evident during a Gram-positive bacterial infection. For that, Gpr43^−/−^ and wild-type mice were infected with *Sp*. After 48 h of pneumococcal infection, we observed an equally high susceptibility of Gpr43^−/−^ mice to infection (sixth day: 40% mortality in WT and 100% mortality in Gpr43^−/−^ mice—data not shown). All together, these data suggest that GPR43 is important to control bacterial infection in the lungs.

### Timely Resolution of Pulmonary Inflammation in Gpr43^−/−^ Mice Was Impaired When Compared to WT Mice after *Kp* Infection

**Figure 2:**
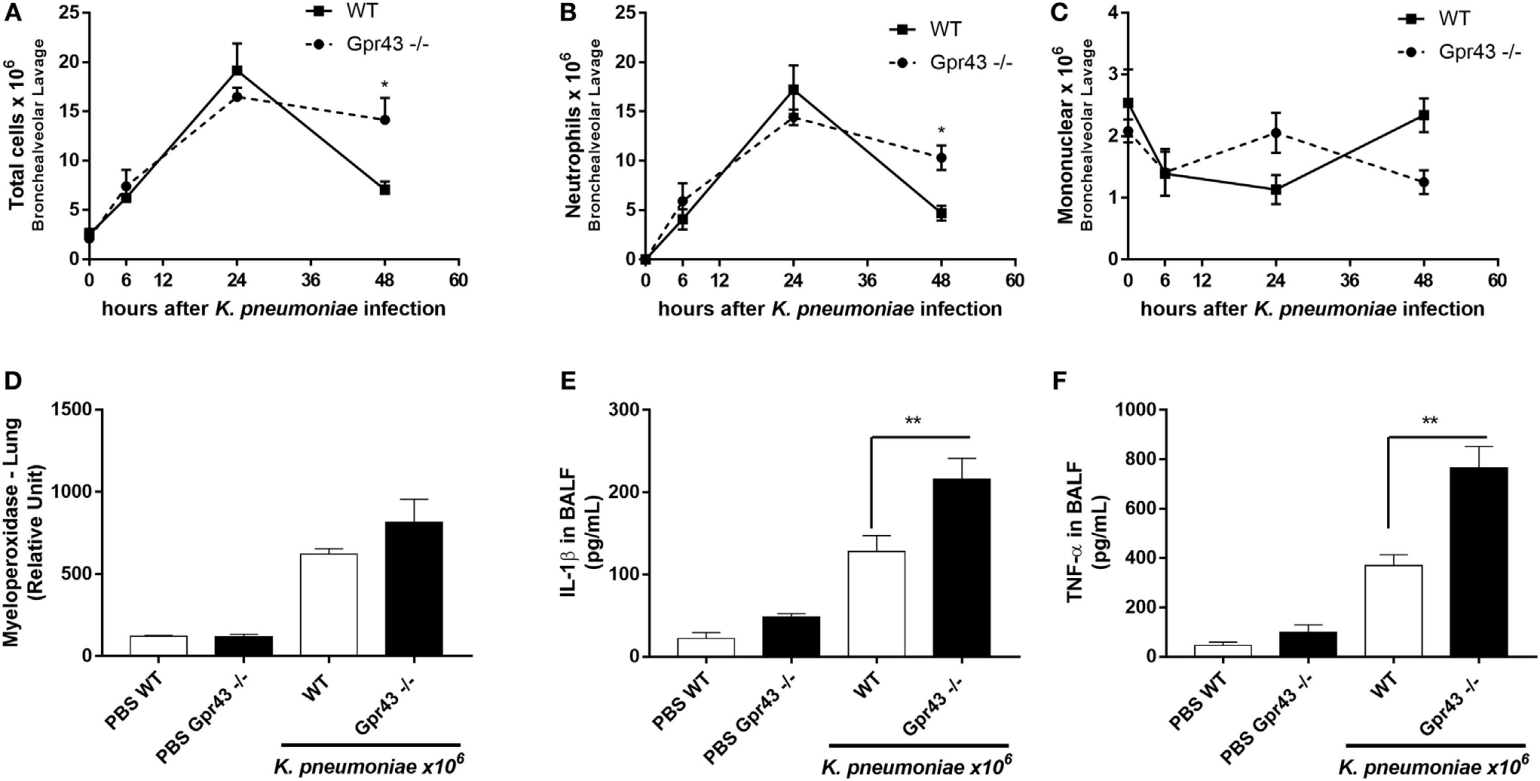
The onset of inflammatory cells in the lung of Gpr43^−/−^ mice 48 h after *Klebsiella pneumoniae* infection. **(A)** Number of total cells infiltration, **(B)** neutrophils, and **(C)** mononuclear cells in airway spaces recovered using bronchoalveolar lavage (BAL) were counted at different time points (0–48 h) after intratracheal inoculation of 1 × 10^6^ CFU of *Kp*. **(D)** Myeloperoxidase activity in homogenized lungs harvested from wild-type and Gpr43^−/−^ mice. **(E)** IL-1β levels and **(F)** TNF-α levels were measured by ELISA in BAL supernatant harvested from wild-type and Gpr43^−/−^ mice 48 h after *Kp* infection. Results are presented as mean ± SEM (*n* = 6). *Statistical difference (*P* < 0.05) when comparing to WT and Gpr43^−/−^ mice. ***Statistical difference (*P* < 0.001) when comparing to WT and Gpr43^−/−^ mice. Results representative of three independent experiments and the statistical (ANOVA—Posttest Newman–Keuls) was used.

Next, aiming to evaluate the inflammatory response that follows *Kp* infection, Gpr43^−/−^ and wild-type mice were injected with 1 × 10^6^ CFU intratracheally, and inflammation was evaluated at different time point. As previously shown (22), *Kp* infection leads to an increased infiltration of leukocytes into the airways and our results showed that there was marked airway inflammatory cells infiltration during the outcome of the infection (Figure 2). There is no difference between Gpr43^−/−^ and wild-type mice at 24 h regarding total cells infiltration (Figure 2A) and neutrophils (Figure 2B). Although wild-type mice seems to exhibited less mononuclear cells compared to GPR43^−/−^ mice at 24 h as observed in (Figure 2C), also, there was no significant difference between them. At 48 h post-*Kp* infection, Gpr43^−/−^ exhibited more total cells infiltration (Figure 2A) and neutrophils (Figure 2B) and less mononuclear cells (Figure 2C) when compared to wild-type mice. However, there were no significant differences in the neutrophils that migrated into the lung tissue measured by the activity of myeloperoxidase (Figure 2D) at 48 h post *Kp* infection. In accordance with marked airway inflammation, the levels of the pro-inflammatory cytokines IL-1β and TNF-α were increased in the BAL when compared to wild-type infected mice (Figures 2E,F). Taken together, these results indicate that inflammation persists longer in Gpr43^−/−^ airspaces, thus Gpr43 plays an important role in the control of inflammation induced by *Kp* infection.

### Oral Treatment with the Gpr43 Metabolic Ligand, Acetate, Ameliorates *Kp*-Induced Pulmonary Inflammation

**Figure 3:**
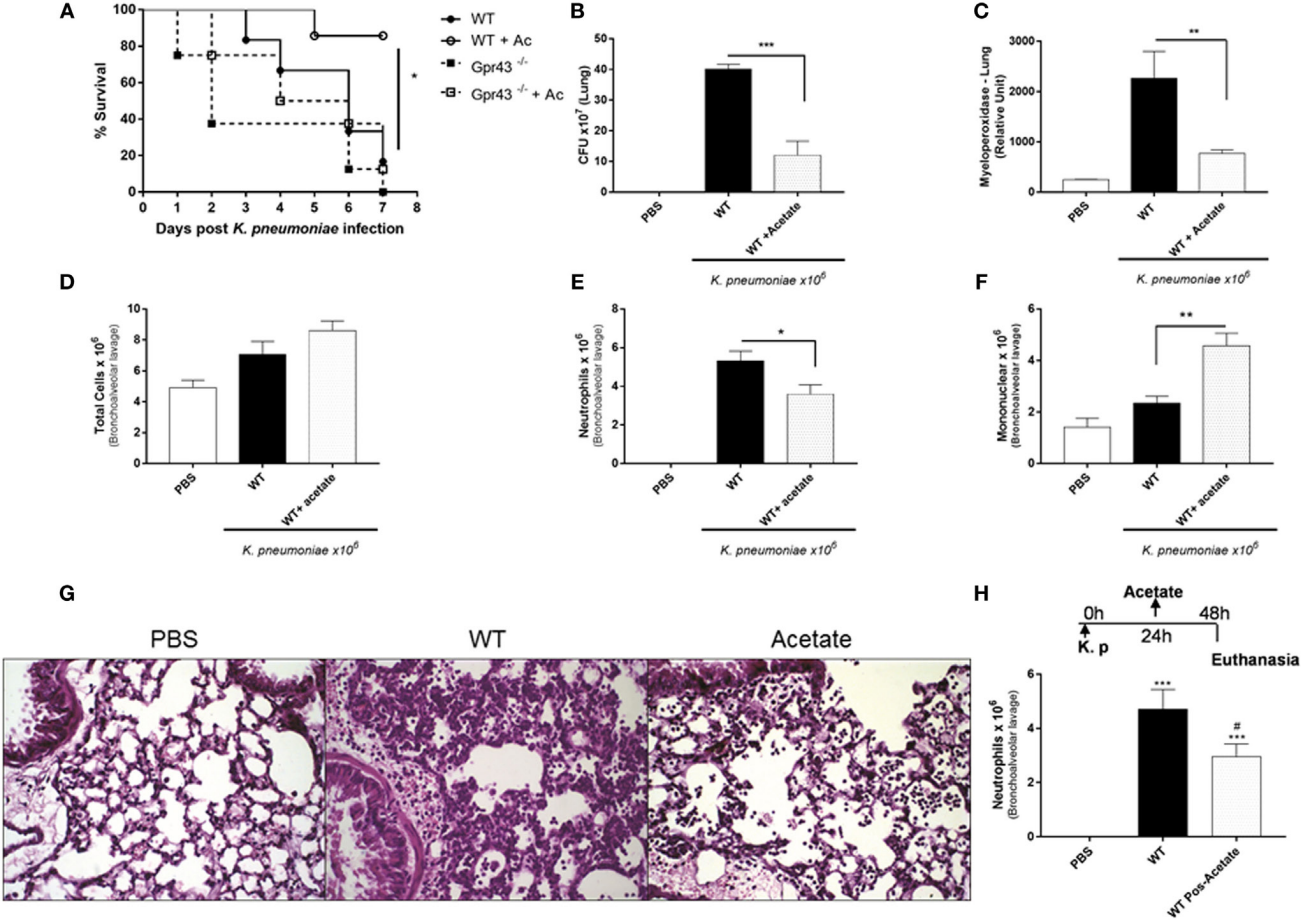
Oral Treatment with the Gpr43 metabolic ligand, acetate in the lung 48 h after *Klebsiella pneumoniae* (*Kp*) infection. **(A)** Survival rate of mice pretreated for 5 days with acetate were observed during 7 days following intratracheal *Kp* (1 × 10^6^ CFU). **(B)** Bacterial burden in the airways was assessed in lung 48 h post-*Kp* infection. **(C)** Myeloperoxidase activity in homogenized lungs harvested from wild-type and wild-type pretreated for 5 days with acetate. **(D)** Number of total infiltrated cells, **(E)** neutrophils, and **(F)** mononuclear cells in airway spaces recovered using bronchoalveolar lavage were counted at 48 h post-*Kp* infection. **(G)** Representative photographs of H&E-stained sections of lung from wild-type mice control [phosphate-buffered saline (PBS)], wild-type mice *Kp* infected, and wild-type pretreated 5 days with acetate *Kp* infected (acetate). Scale bar—400 mm. **(H)** Number of neutrophils from posttreatment mice with acetate (150 mM) by gavage 24 h after *Kp* infection. Results are expressed as mean ± SEM of 8–10 mice per group. *Statistical difference (*P* < 0.05) when comparing to WT and WT + acetate. ***Statistical difference (*P* < 0.001) when comparing to WT and WT + acetate. Results representative of three independent experiments and the statistical (ANOVA—Posttest Newman–Keuls) was used.

Because acetate is the mostly abundant microbial metabolite produced which is also able to reach systemic levels and to bind to Gpr43, we pretreated mice with acetate in drinking water (150 mM) for 5 days and then infected with *Kp*. We observed that acetate treatment was able to reduce the mortality and bacterial counts (Figures 3A,B). Mononuclear cells were increased in the airway space in acetate-treated mice 48 h post-*Kp* infection (Figure 3F). Neutrophil infiltration is a sign of inflammation, and neutrophil clearance is the most important marker of recovery from inflammation. Accordingly to this, we observed a reduction in myeloperoxidase activity in the lungs and neutrophils in the airway space (Figures 3C–E) with no alteration in the total number of cells after acetate treatment. Acetate was able to protect lungs from pathological injury observed by reduced pulmonary inflammatory cell infiltration in lung parenchyma (Figure 3G). Furthermore, we also treated mice with acetate (150 mM—oral gavage) 24 h after infection and it was also able to reduce neutrophils in the airway space (Figure 3H). In accordance with previous results, oral treatment with acetate reduces TNF-α and IL-1β levels in lungs 48 h after *Kp* infection (Table 1). These data corroborate with the Gpr43^−/−^ result, suggesting the role of acetate/Gpr43 activation in the control of inflammation induced by *Kp* infection.

**Table 1:** Cytokine levels in the lung of mice.

	Phosphate-buffered saline	WT	WT + Acetate
TNF-α (pg/ml)	1,948.71 ± 123.6	3,787.36 ± 187.0	2,078.87 ± 589.1*
IL-1-β (pg/ml)	663.82 ± 27.72	3,829.19 ± 396.19	2,261.96 ± 833.55*

*Statistical significance. P < 0.05.

### Macrophages from Gpr43^−/−^ Mice Had Impaired Phagocytosis, and *In Vivo* Transference Restores the Protective Phenotype

**Figure 4:**
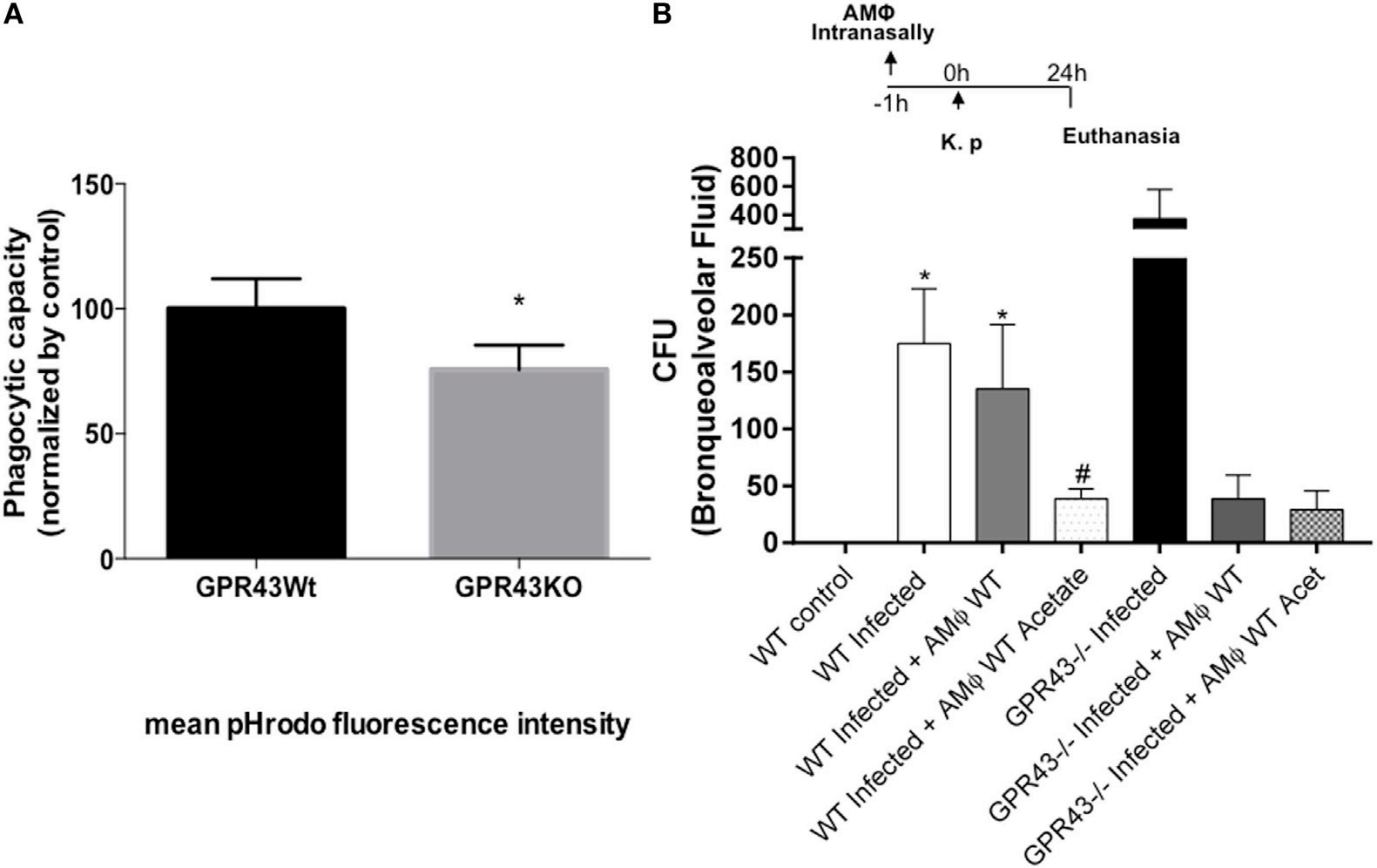
Macrophages from Gpr43^−/−^ had impairment in phagocytosis and alveolar macrophages (AM) transference *in vivo* restore protective phenotype. **(A)** Macrophages from wild-type and Gpr43^−/−^ were incubated with opsonized-pHRodo-marked *Kp* (MOI 1:1) for 60 min. Samples were analyzed by flow cytometer, and the mean fluorescence of bacteria within the cells were analyzed. **(B)** Bacterial burden in the airways was assessed in bronchoalveolar fluid 24 h post-*Kp* infection. Results are presented as mean ± SEM (*n*=6). *Statistical difference (*P* < 0.05) when comparing to WT control (ANOVA—Posttest Newman–Keuls). ^#^Statistical difference (*P* < 0.05) when compared with WT infected (ANOVA—Posttest Newman–Keuls). Results representative of three independent experiments.

To gain further insight into the possible mechanism by which Gpr43 are involved in the protection of *Kp* infection, we analyzed the capacity of AMs to phagocytose bacteria. Resident AMs are the first line of lung defense against respiratory pathogens without evoking a measurable systemic inflammatory response. Because MF have been shown to be important during *Klebsiella*-induced pneumonia (22), we decided to investigate the contribution of Gpr43^−/−^ MF into their susceptible phenotype. We observed that MF from Gpr43^−/−^ mice had impaired phagocytosis when compared to the wild type (Figure 4A). Therefore, this might be a possible mechanism of increased bacterial burden in Gpr43^−/−^ mice. To prove this hypothesis, we transferred *in vivo* AM from wild-type mice to Gpr43^−/−^ mice, intranasally, 1 h before the *Kp* infection. The AMs from wild-type mice restored protective phenotype on Gpr43^−/−^, showing a reduction of bacterial burden in the airways. Interestingly, AM recovered from acetate-treated WT mice reduced bacteria numbers on WT mice. Our results suggest the importance of Gpr43 in the clearance of bacteria (Figure 4B).

### Neutrophils from Gpr43^−/−^ Mice Had Impaired Chemotaxis, Phagocytosis, and Cytokine Production

**Figure 5:**
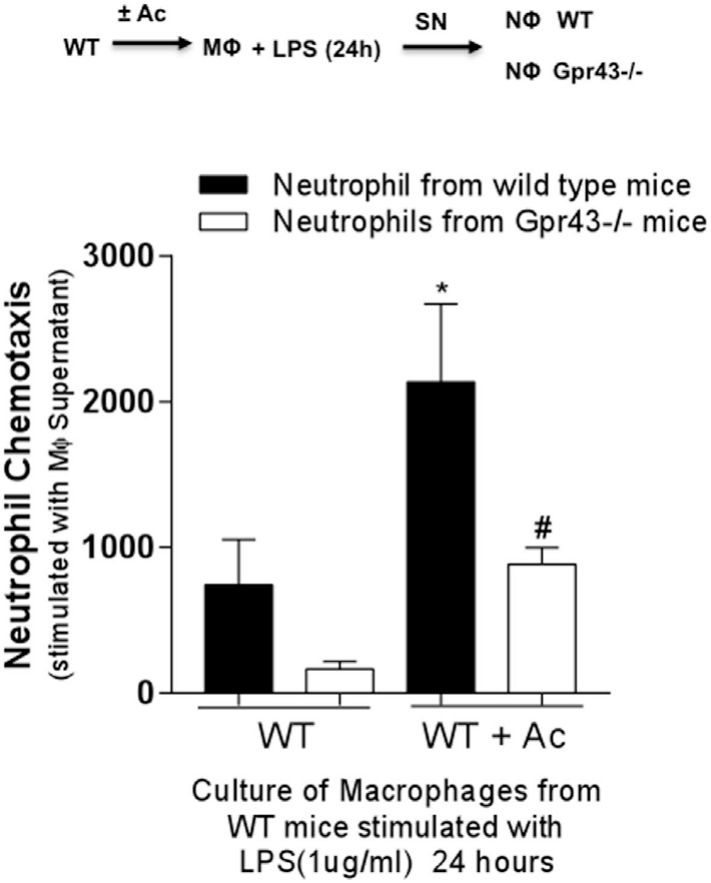
Neutrophils from Gpr43^−/−^ mice present less chemotaxis. Peritoneal macrophage (MF) harvest from wild-type mice treated or not with 150 mM of acetate was stimulated with lipopolysaccharides (LPS) for 24 h. The supernatant MF culture was used as a chemoattractant to bone marrow neutrophils elicited from wild-type and Gpr43^−/−^ mice. Results are presented as mean ± SEM (*n*=6). *Statistical difference (*P* < 0.05) when compared with WT (ANOVA—Posttest Newman–Keuls). ^#^Statistical difference (*P* < 0.05) when compared with WT + Acetate (ANOVA—Posttest Newman–Keuls). Results representative of three independent experiments.

**Figure 6:**
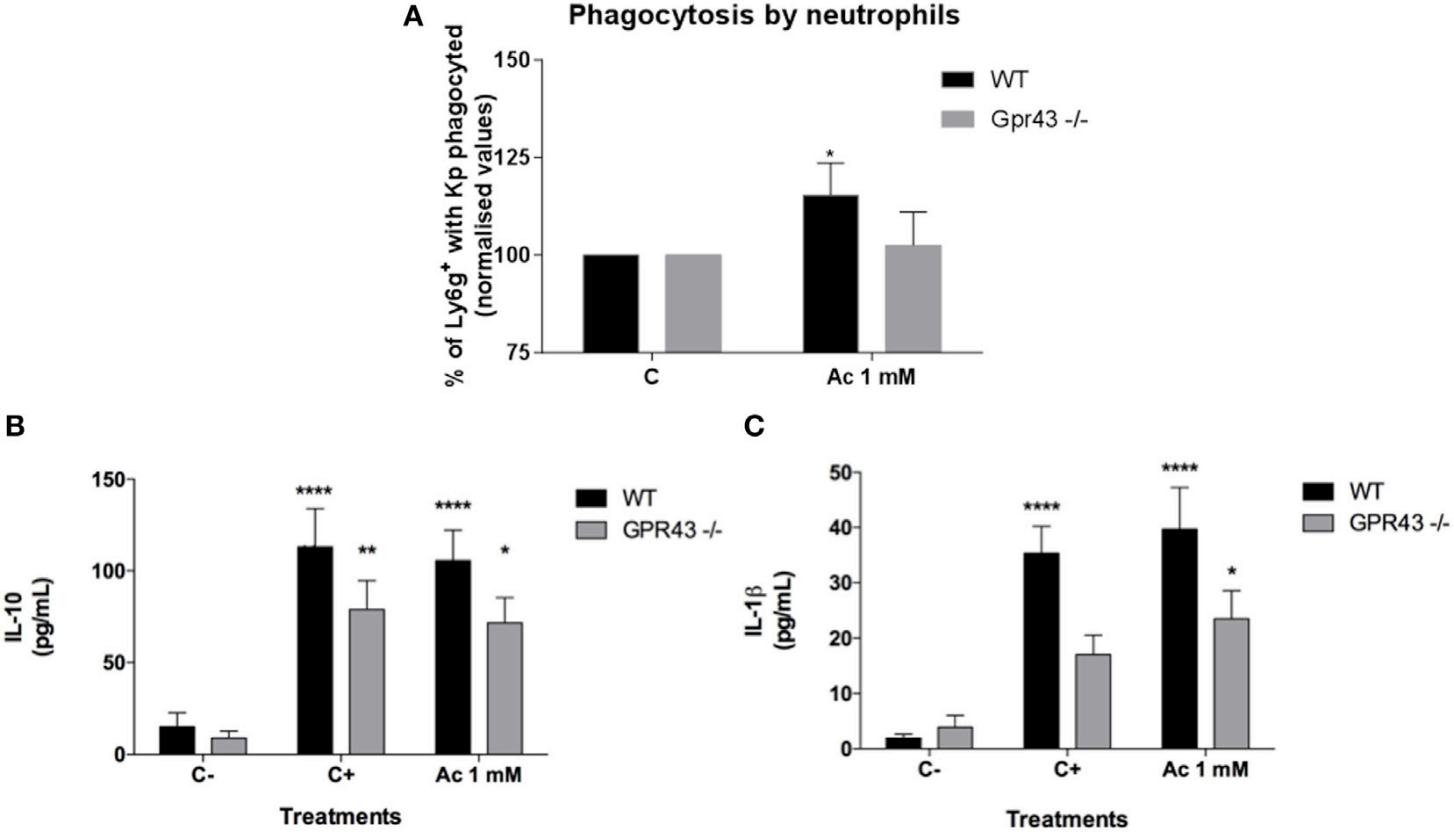
*In vitro* neutrophils analyses of phagocytosis and cytokines production after *Klebsiella pneumoniae* (*Kp*) exposure. **(A)** Bone marrow neutrophils from wild type and Gpr43^−/−^ were incubated with opsonized-pHRodo-marked *Kp* (MOI 1:1) and acetate (1 mM) for 120 min. Samples were analyzed by flow cytometer, and the mean fluorescence of bacteria within the cells were analyzed. **(B)** IL-10 and **(C)** IL-1β were measured in supernatant from neutrophils by ELISA. Results are presented as mean ± SEM (*n*=6). C− = without bacteria C+ = with bacteria. *Statistical difference (*P* < 0.05) when compared with WT control (ANOVA—Posttest Newman–Keuls). **Statistical difference (*P* < 0.01) when compared with WT control without bacteria (ANOVA—Posttest Sidak’s). Results are representative of three independent experiments.

It was observed that Gpr43^−/−^ mice present a marked lung inflammatory neutrophil infiltration; however, the role of neutrophils during *Kp* infection was not yet fully investigated. In order to investigate the role of Gpr43^−/−^ in neutrophils, resident MF from wild-type and Gpr43^−/−^ pretreated or not with oral acetate were stimulated with LPS for 24 h. The supernatant was used as a chemoattractant to the elicited bone marrow neutrophils. Neutrophils from Gpr43^−/−^ mice showed reduced migration when compared to the wild-type mice and the treatment with acetate was able to increase neutrophils migration from WT mice, which was not observed in GPR43^−/−^ mice (Figure 5). This result show that GPR43 receptor expressed on neutrophil’s surface played an important role in neutrophil activity state and subsequent migration. In order to better understand the role of GPR43 in neutrophils, we analyzed their capacity to phagocytose bacteria. As observed in MF, neutrophils from Gpr43^−/−^ mice also show reduced capacity to phagocytose bacteria when compared to the wild type (Figure 6A). These results are accompanied with no significant difference in cytokine levels between wild-type and Gpr43^−/−^ mice (Figures 6B,C).

## Discussion

The importance of gut microbiota mediating several features of gut immune responses has been appreciated since the past decade. More recently, the relationship between gut microbiota and the lungs (the called “gut–lung axis”) has also been addressed (25–27). Both lungs and gut have an immense surface area and are exposed to several allergens and pathogens on a daily basis. Therefore, both organs have important barriers against harmful particles or pathogens. One of those barriers is the presence of indigenous microorganisms, which are important for both host physiological and defense functions. Microbiota metabolites such as SCFA (acetate, propionate, and butyrate) are important for metabolic functions in the gut but also have been shown to interfere with immune responses, modulating inflammatory responses (11, 14, 28). Pneumonia induces disbiose and reduced levels of SCFA. SCFA are sensed by GPRs receptors, such as GPR43 and GPR109, leading to activation of signaling pathways in immune or epithelial cells (12, 13, 21).

GPR43 is the leading receptor for acetate and has been shown to control responses such as adipogenesis and modulation of gout inflammation (13, 29). Here, we highlighted the importance of the microbial metabolic sensor Gpr43 and its ligand acetate as modulators of lung innate immunity against bacterial pneumonia. In the present study, we have shown for the first time that Gpr43 KO mice exhibit: (1) increased susceptibility to *Kp* lung infection, which was related to an (2) increased proliferation of bacteria in the airways of mice and uncontrolled inflammatory responses. The permanence of neutrophils in the lung resulted in greater lung damage following infection, with increased histological score. In addition, (3) GPR43 expression in neutrophils and MF was important for the phagocytosis and for bacterial killing. Finally, we showed that the (4) acetate/GPR43 pathway is crucial during infection by promoting pathogen clearance and host recovery. SCFA mainly produced by gut commensal bacteria were shown to increase production of ROS in inflammatory cells contributing for an effective immune response, but importantly they have also been shown to play a role in the resolution of inflammation, by leading to apoptosis of neutrophils, production of anti-inflammatory cytokines, and restoration of tissue homeostasis (11, 28).

It is known that during pneumonia, an unregulated inflammatory response can lead to intense lung injury, loss of function, and poor prognosis. In this regard, we observed that GPR43 absence resulted in increased influx and persistence of neutrophils, which supports the increased levels of IL-1β. IL-1β production is an important cytokine mediating crucial immune responses against *Kp* (30); therefore, higher levels of this cytokine in Gpr43^−/−^ mice could be contributing to the increased pulmonary tissue injury. Interestingly, despite the greater inflammatory response in GPR43-deficient animals, an increased number of bacteria was observed in the lung of those animals in comparison with wild-type mice. Therefore, instead of dealing with the pathogen, the increased inflammatory response contributed to the pathology observed in the lungs. Aiming a better understanding over the uncontrolled proliferation of bacteria in the airways of Gpr43^−/−^ mice, we showed that the functions of neutrophils and MF (phagocytosis of bacteria) were impaired as demonstrated by the *in vitro* data. Altogether, GPR43 plays a crucial role in both modulation of inflammation and control of pathogen burden. Indeed, we and others have shown that the signaling triggered by acetate-GPR43 in inflammatory cells leads to increased production of ROS, activation of inflammasome, increased phagocytosis, and killing of bacteria (13, 23). On the other hand, GPR43 signaling is also important to balance inflammation leading to a mild recruitment of neutrophils and production of pro-resolving mediators. In this regard, it culminates with an effective clearance of bacteria and reduction of the collateral lung injury. In agreement with our results, the acetate/Gpr43 pathway was shown to promote resolution of inflammation in an experimental model of gout (28) and was also shown to be important to prime germ-free (GF) mice’s MF, improving their ability to respond to a sterile insult (13). Since long-lasting inflammatory responses can lead to severe lung injury, timely resolution of the inflammation is critical for the host to minimize tissue damage and to regain homeostasis (31).

*Klebsiella pneumoniae*, an opportunistic Gram-negative pathogen, triggers an important inflammatory response in the lungs mainly due to the recognition of membrane LPS. Here, we have showed that MF harvested from acetate-treated mice and exposed to LPS could induce higher chemotaxis of neutrophils from WT mice than of MF isolated from non-treated mice. Importantly, this phenomenon was dependent on GPR43 since neutrophils derived from GPR43-deficient mice did not migrate as much as the wild-type cells did. Therefore, it suggests that acetate priming of peritoneal MF is important to prompt an initial inflammatory response and neutrophil recruitment into the lungs. These results reinforced the dual role of GPR43 in mediating the proper immune response against *Kp*. We hypothesize that, in early time points, acetate stimulates neutrophils recruitment and activation *in vivo* assuring the killing of bacteria, and on the other hand, later during the infection, acetate contributes to the resolution of pulmonary inflammation/injury probably by diminishing neutrophil survival by the increase of IL-10 production (22).

The concept of the host microbiota being pivotal to the maturity and function of the immune system, especially assuring host defense against pathogens is well appreciated in the literature. GF mice display a significantly different inflammatory responsiveness pattern when compared to conventional mice, being hyporesponsive to infections (9, 26).

Notably, several works have described the role of gut microbiota to improve lung defenses by directly activating local cells thought PRR signaling by LPS or PSA components, for example (9, 10, 26). Despite this, gut microbiota disruptions due to dietary or to antibiotics treatment have also been associated to pulmonary diseases suggesting that microbiota composition plays an important role to lung immune responses (19, 32, 33). The intestinal microbiota exhibits dynamic responses to external stimulation (i.e., diets). Poor quality diets or an unbalanced energy that can lead to malnutrition have been described to lead to changes in the gut microbiota composition (34, 35) that can influence disease progression and abnormalities of the leukocytes, impairing the proper defense against infections (36, 37). Indeed, malnutrition has been known as the main contributor to deaths from infections, even after HIV burden (38). In this regard, malnutrition is one of the leading risk factors to the development of pneumonia. Moreover, malnourished patients are more likely to die from pneumonia than well-nourished ones (39). Recently, Desai and collaborators (40) have described that low fiber dietary was related to functional changes associated to intestinal dysbiosis and host susceptibility to intestinal infection. Furthermore, of equally importance, dietary fiber supplementation and SCFA, on the other hand, have been shown to protect mice not only by promoting host intestinal homeostasis but also against pulmonary inflammation in allergic diseases (19, 21). These findings are particularly relevant to our own regarding to the fact that deficiency in the microbial metabolic sensor GPR43, which recognizes acetate produced by microbiota fermentation of fiber, increased susceptibility to *Kp* infection.

Herein, we hypothesize that Gpr43-dependent effect of SCFA during bacterial pulmonary infections leads to a controlled inflammatory response. This raises the idea that GPR43 may be a relevant receptor that interfaces the crosstalk between host health nutritional state and lung immunity by intestinal microbiota energy source.

Moreover, once intestinal microbiota, especially acting through production of active metabolites, dictates the host ability and resistance against pathogens, interfering with gut microbiota or given probiotics might provide new perspective and approaches to pulmonary infections. In this regard, a recent clinical trial reported that probiotic therapy using the bacterial strain *Lactobacillus plantarum* prevented infections, respiratory ones included, in newborns (41). Also, our group has already shown in a murine experimental model of pneumonia that treatment with the commensal bacteria *Bifidobacterium longum* 5^1A^ protected mice from *Kp* lung infection (22). This effect was in part due to the modulation of AMs activity though induction of ROS and of increasing the bacterial killing. Of note, these effects were amplified by the use of live probiotics. *B. longum* strain is well known characterized to be the most important bacteria that produce acetate, reinforcing thus our hypothesis that acetate produced by commensal bacteria may modulate the lung immune response against respiratory infections.

In conclusion, we report a previously unappreciated role of GPR43 in promoting host timely inflammation resolution of Gram-negative bacteria-induced lung injury. In addition, our study demonstrates that acetate-binding GPR43 can be critical for recovery from *Kp*-induced lung injury by modulating neutrophils and MF activity. Interventions that improve SCFA levels by gut microbiota may promote resolution of inflammation.

## Ethics Statement

We hereby certify that the Protocol no 219/2014, related to the project entitled “Study of inflammatory response in a experimental infection model by the bacterial pneumoniae,” under supervision of Mauro Martins Teixeira, is in agreement with the ethical Principles in Animal Experimentation, adopted by the Ethics comminttee in Animal Experimentation (CEUA/UFMG) and was approved in 24/09/2014. This certificate expires in 23/09/2019.

## Author Contributions

AV, LT, and IG analyzed data and wrote the paper. AV, LT, IG, RC, JF, VR, CG, MR, CF, and MV performed experiments and analyzed data. GC performed histological analysis. SO, FM, MT, CM, MV, and AV provided expertise and improvements in the issue and helped with the paper discussion. AV designed research.

## Conflict of Interest Statement

The authors declare that the research was conducted in the absence of any commercial or financial relationships that could be construed as a potential conflict of interest.
